# Development, implementation, and evaluation of a rapid response system at a Nigerian teaching hospital, a novel idea in sub-Saharan Africa

**DOI:** 10.3389/fmed.2025.1583470

**Published:** 2025-07-09

**Authors:** Promise Ariyo, Seung W. Lee, Asad Latif, Chinyere Egbuta, Vinciya Pandian, Olufemi Bankole, Ibironke Desalu, John Sampson, Bradford Winters

**Affiliations:** ^1^Department of Anesthesiology and Critical Care Medicine, Johns Hopkins Hospital, Baltimore, MD, United States; ^2^Department of Anesthesiology, Critical Care and Pain Medicine, Boston Children’s Hospital, Boston, MA, United States; ^3^School of Nursing, Johns Hopkins University, Baltimore, MD, United States; ^4^Department of Surgery, University of Lagos College of Medicine, Lagos, Lagos, Nigeria; ^5^Department of Anesthesiology, University of Lagos College of Medicine, Lagos, Lagos, Nigeria

**Keywords:** Pilot hospital-based, multidisciplinary, rapid response system, failure to rescue

## Abstract

**Aim:**

Little is known about the incidence of clinical deterioration and cardiopulmonary arrest (CPA) on general hospital units in low-and middle-income countries (LMICs) or how rapid response systems (RRSs) might impact these events. Implementation of RRSs in high-income countries has been shown to reduce the incidence of CPA and mortality. The aim of this study was to determine whether implementation of an RRS is feasible in an LMIC medical center.

**Methods:**

We developed and implemented an RRS in a large academic medical center in Lagos, Nigeria, in three phases: (1) Needs assessment and stakeholder engagement, (2) Infrastructure setup and education, and (3) Implementation and data collection. We collected data on incidence of rapid response events, attendance ratio and time of arrival of the designated clinical staff, triggers for the rapid response calls and common interventions at the events.

**Results:**

Over the 7 months study period, 997 patients were admitted to the intervention-eligible units, and 95 RRS events occurred in 55 patients. In 11 RRS activations (11.6%), no rapid response team member responded. Anesthesia residents attended 73.7% of the events, and anesthesia techs and nurses attended roughly 38% each. Internal medicine residents responded to 13.7% of RRS activations. The average time to arrival was 13 min. The most common trigger was altered mental status, followed by hypoxia and hypotension. Seventy-six percent of patients survived their initial RRS activation, and 83% died while in hospital. Common interventions were vasopressor use, oxygen supplementation, and intravenous fluid administration. No patient was transferred to the designated intensive care unit after an RRS activation owing to lack of beds. Six patients were transferred to the makeshift ICU, all of which required vasopressor support.

**Conclusion:**

While barriers remain, the development and implementation of an RRS program in an LMIC medical center is feasible.

## 1 Introduction

Clinical deterioration resulting in cardiopulmonary arrest (CPA) among hospitalized patients on general hospital units (also known as failure to rescue) is associated with very high mortality and has best been described in high-income countries (1–4). An extensive body of literature demonstrates that in high-income countries, clear warning signs often precede deterioration to CPA, leaving substantial time to intervene (5, 6). Data for low- and middle-income countries (LMICs) are scarce but suggest an unacceptably high incidence of CPAs, many of which are not witnessed (7). Given that LMICs account for nearly 90% of all world-wide trauma-related deaths, maternal deaths, and deaths from overwhelming infections (5, 8, 9), we suspect that unrecognized clinical deterioration on general units in these countries contributes substantially to these high rates of adverse outcomes.

Rapid response systems (RRSs) were developed in high-income countries as a patient- safety intervention to address this dangerous problem (9, 10). At its core, an RRS includes an afferent component that establishes processes and criteria for identifying patients who are deteriorating or at risk of deteriorating and activating a response team of clinicians that forms the efferent component. Fully mature RRSs also include administrative components, education components, and components for collecting and analyzing data that are used for ongoing quality improvement and regulatory requirements.

The rapid response team is typically activated by single physiologic or laboratory data thresholds or amalgamated and weighted scores based on these data, as well as team or family member concern. The responding team is a multidisciplinary group of clinicians that may include physicians, nurses, anesthesia techs, pharmacists, and others, depending on local resources and staffing availability. This team may also proactively evaluate high-risk general unit patients (known as critical care outreach) and educate and act as a liaison to unit staff. Various RRS models have been implemented in many developed countries over the last 20–25 years as a potent patient-safety intervention. In the United States, they were endorsed by the Institute for Healthcare Improvement’s “100,000 Lives Campaign” in 2005 and then required by the American Joint Commission for Hospital Accreditation as part of the Commission’s Patient Safety Goal for 2009. Such programs have reduced unanticipated CPA and in-hospital mortality in developed countries for both adult and pediatric patients (9, 10).

Patient safety and mitigating preventable harm have become a global health priority; however, RRSs have not been fully evaluated in low-resource settings where the need is arguably the greatest. One study from an LMIC in South Asia demonstrated RRS effectiveness in reducing CPA and mortality (11). Such programs could have tremendous impact in sub- Saharan Africa. Because the hospitalized patients there are typically younger, often with relatively few comorbidities, well-orchestrated and timely response to acute deterioration could potentially prevent complete decline and CPA (12). Such programs may also serve as an indicator for the readiness of local healthcare systems in providing effective emergency, essential surgical, and anesthesia care as components of universal health coverage, as urged by the 68th Assembly of the World Health Organization (13).

Additionally, the implementation of RRSs may be especially beneficial in sub-Saharan Africa, where critical care facilities are scarce despite the greater burden of critical illness (14). For example, Uganda has one intensive care unit (ICU) bed per million population (8). Nigeria only has 380 critical care nurses for a population of 140 million, compared with more than 500,000 critical care nurses in the United States for a population of 324 million (15, 16). Therefore, complex patients are cared for on the general hospital unit, adding strain to overworked and under-resourced nurses. As a result, inadequate patient assessment and monitoring, inappropriate treatment, and communication breakdown contribute to poor outcomes in LMICs (17). Moreover, the implementation of RRSs could improve patient-safety culture through improved critical care training, care coordination, and earlier identification of deterioration trends that illuminate faulty processes.

In this report, we describe development and implementation of an RRS at Lagos University Teaching Hospital (LUTH), the effects of the RRS on the quality of critical care on general units and lessons learned about RRS implementation in similar contexts.

## 2 Materials and methods

### 2.1 Setting

Lagos University Teaching Hospital is a large tertiary teaching hospital in Nigeria. It is a government hospital with 761 beds and a six-bed ICU. The ICU is managed by anesthesiologists and patients on the floor units are managed by internal medicine doctors and surgeons. Nigeria is an LMIC in West Africa, with a total population of ∼ 182 million. The mortality rate of Nigerians adult males is 356 per 1,000 male adults https://data.worldbank.org/indicator/SP.DYN.AMRT.MA?locations . We established an academic partnership and collaboration between LUTH team members in Lagos, Nigeria, and the Johns Hopkins University School of Medicine in Baltimore, MD, United States, to develop and implement an RRS at LUTH. The project occurred in three phases: (1) Needs assessment and stakeholder engagement, (2) Infrastructure setup and education, and (3) Implementation rollout and data collection. We prospectively collected data on patient demographics and clinical outcome during the implementation. We had IRB approval from both Johns Hopkins University and LUTH.

### 2.2 Phase 1: needs assessment and stakeholder engagement

Investigators from Johns Hopkins traveled to Nigeria for an introductory meeting with hospital administrators and department heads. Participants included leadership in the departments of nursing, anesthesiology, surgery, internal medicine, and pharmacy, as well as the chief residents in these specialties. We jointly reviewed their existing practice of identifying and triaging high-acuity patients and the potential failure modes and deficiencies in their practice and processes. We subsequently discussed the proposed project and its potential usefulness in improving the delivery of quality care to the patients at LUTH. We jointly identified potential hurdles to successful implementation, including perceptions of increased work burden, lack of standardized triggers for rapid response calls, poor organization of command chains, poor communication infrastructure, poor access to emergency medications and clinical monitors, and limitations in the number of ICU beds. We worked with staff and local vendors to address some of the potential obstacles.

### 2.3 Phase 2: infrastructure setup and education

#### 2.3.1 Communication tools

We worked with a local telecommunications company to create stationary phones on the study units that were designed to call mobile “rapid response phones” with the push of one number. This call was intended to activate the mobile phones that would be carried by members of the rapid response teams.

#### 2.3.2 Crash carts and monitors

We worked with the nursing team and pharmacists to update the crash carts with essential supplies and medications, including advanced cardiac life support medications. Importantly, patients were to be charged for the medications after administration in an emergency setting, as opposed to the existing practice of having family members purchase the medications from the pharmacy before use, a very time-inefficient and costly practice.

#### 2.3.3 Assessment tools

**FIGURE 1:**
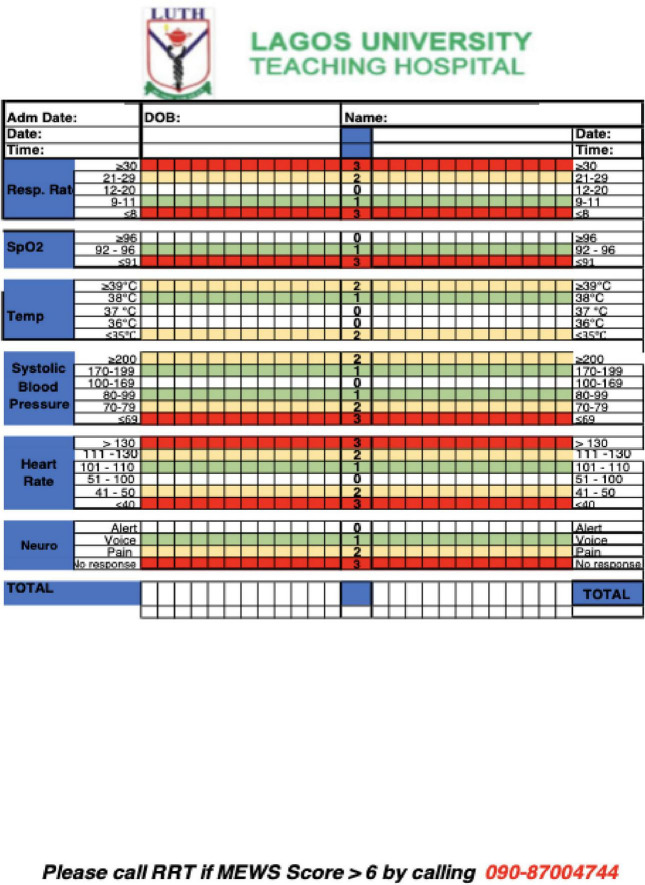
Modified Early Warning Scores (MEWS) used to determine when the rapid response system should be activated (21).

The research team and the providers at LUTH endorsed the use of the Modified Early Warning Score (MEWS) protocol as an identification tool (18, 19). MEWS is a vital sign–driven protocol that amalgamates data into a weighted score for predicting deterioration risk and is used by bedside nurses to identify at-risk patients (Figure 1) (20).

Nurses were then empowered to activate the RRS team with the established protocol.

#### 2.3.4 Rapid response team (RRS efferent component)

Rapid response teams consisted of two providers (an anesthesia resident and an internal medicine resident), a senior ICU nurse with the expertise and clinical flexibility to attend remote events and an anesthesia technician. The anesthesia technician functioned both in and out of the operating room as airway support personnel, facilitating access to airway equipment, and providing technical assistance for airway support among their other duties. Rapid response team members had a weekly schedule, and each carried a dedicated RRS mobile phone while on call.

#### 2.3.5 The study units

Two intervention units were identified based on perceived readiness for culture change (one medical unit and one surgical unit). The study team spent time in these units to observe current practices, engage the local staff, and identify a local champion. In each intervention unit, a crash cart was positioned close to the nursing station, along with a makeshift, temporary ICU bed that could be used until a dedicated ICU bed became available or until the patient improved sufficiently to return to their regular unit bed. The decision to use the makeshift ICU bed would be at the discretion of the rapid response teams. Colorful paper copies of the MEWS tool sheet were available on the units and attached to each patient’s chart for easy access (Figure 1). Two control units were identified for comparison purposes.

#### 2.3.6 Campus-wide education and introduction of the project

We embarked on intensive training both for the frontline providers who were involved in the identification of at-risk patients and for responding providers. Designated members of the research team participated in a campus-wide multidisciplinary grand rounds with over 100 attendees. We delivered more focused training to the providers and nursing staff who would eventually constitute the rapid response teams.

The bedside nurses (afferent limb) received an online training course on the MEWS tool that would be used to identify at-risk patients. The house staff on the response teams (efferent limb) were trained in the Fundamentals of Critical Care Support, a course designed by the Society of Critical Care Medicine to train non-intensivists to identify and manage critically ill patients pending appropriate critical care consultation^1^. The nursing staff and anesthesia technicians who were part of the efferent limb of the RRS were also engaged in intensive nursing critical care courses before implementation.

### 2.4 Phase 3: implementation and data collection

We implemented the RRS in January 2019 and collected data through July 2019. We collected data on patients admitted to the intervention units and control units by using prospective chart reviews that included data on patient demographics, admission diagnosis, comorbid conditions, hospital length of stay, and mortality.

On a bimonthly basis, we provided motivational lunches and talks by inspirational guest speakers. We gave awards to the most “responsive resident” and “best RRS nurse,” and monthly “Nurse Angel” awards. In addition, rapid response team T-shirts were used to motivate and encourage the residents and nurses.

All RRS calls were reviewed during a monthly meeting of stakeholders from the various departments. Data obtained from the data collection tool were collated, and successes and barriers were discussed and recorded. Improvement plans were put in place regularly. We defined feasibility as having at least two members of the efferent team respond to RRS event more than 50% of the time.

## 3 Results

**FIGURE 2:**
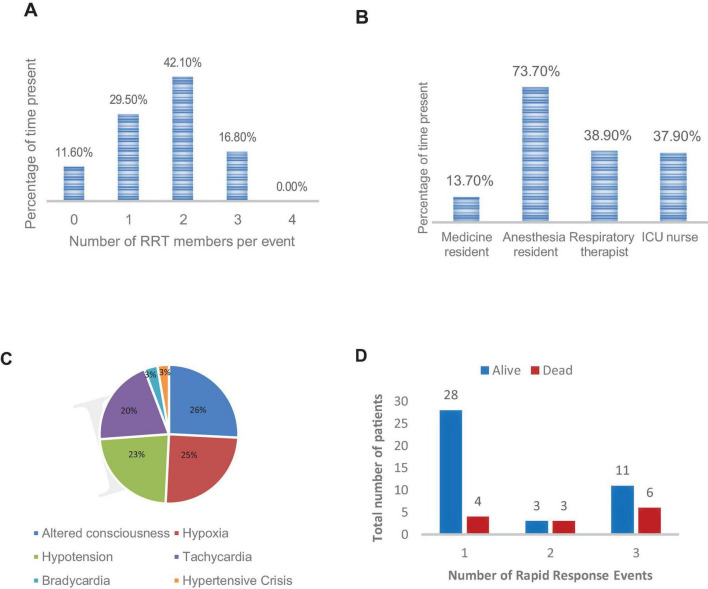
Characteristics of rapid response team activations. **(A)** Number of rapid response team members per event. **(B)** Rapid response attendance by role. **(C)** Indications for rapid response team activation. **(D)** Rapid response team event number stratified by outcome. ICU, intensive care unit; RRT, rapid response team.

The RRS was implemented over a 7 months period during which 577 patients were admitted to the intervention units and 420 to the control units (Table 1). A total of 95 events in the intervention units prompted activation of the RRS team. Eighty-four (88%) of these activations resulted in at least one team member response to the activation. In 11 RRS activations (11.6%), no rapid response team member responded. None of the RRS events had all four team members present (Figure 2A). Anesthesia residents were the most frequent responders, arriving at 70 events (73.7%), followed by anesthesia techs and nurses with ∼38% attendance each (Figure 2B). The average time to arrival of any rapid response team member was 13 min. The clinicians that missed the rapid response reported being occupied with other clinical responsibilities at the time of the events.

**TABLE 1:** Baseline characteristics for patients admitted to intervention units from January 2019 through July 2019.

Characteristics	Categories	Survival group (*n* = 474)	Mortality group (*n* = 103)	*P*-value
Age, years, median (IQR)	–	45 (34–60)	56 (44–49)	0.001
Age, *n* (%)	18–44	209 (44.1)	28 (27.2)	0.002
	45–64	166 (35.0)	40 (38.8)	
	> 64	99 (20.9)	35 (34.0)	
Sex, *n* (%)	Male	246 (51.9)	56 (54.4)	0.759
	Female	228 (48.1)	47 (45.6)	
Diagnosis, *n* (%)	Infection	88 (18.6)	20 (19.4)	0.000 (*p* < 0.001)
	Neoplasm	60 (12.7)	33 (32.0)	
	Neurologic	54 (11.4)	5 (4.9)	
	Cardiac	64 (13.5)	16 (15.5)	
	Other	208 (43.8)	29 (28.2)	
Pre-existing conditions, *n* (%)	DM	81 (17.1)	14 (13.6)	0.559
	CKD	35 (7.4)	8 (7.8)	1.000
	HIV/AIDS	24 (5.1)	9 (8.7)	0.091
	HTN	183 (38.6)	37 (35.9)	0.692
LOS, days, median (IQR)	–	9 (5–15)	10 (4.5–19)	0.002
**Characteristics**	**Categories**	**Survival group (*n* = 351)**	**Mortality group (*n* = 69)**	***P*-value**
Age, years, median (IQR)	–	46 (37–60)	44 (37–65)	0.421
Age, *n* (%)	18–44	152 (43.3)	35 (50.7)	0.033
	45–64	137 (39.0)	16 (23.2)	
	> 64	62 (17.7)	18 (26.1)	
Sex, *n* (%)	Male	164 (46.7)	32 (46.4)	1.000
	Female	187 (53.3)	37 (53.6)	
Diagnosis, *n* (%)	Infection	74 (21.1)	18 (26.1)	0.085
	Neoplasm	57 (16.2)	17 (24.6)	
	Neurologic	79 (22.5)	7 (10.1)	
	Cardiac	20 (5.7)	2 (2.9)	
	Other	121 (34.4)	25 (36.2)	
Pre-existing conditions, *n* (%)	DM	72 (20.5)	8 (11.6)	0.194
	CKD	22 (6.3)	4 (5.8)	0.735
	HIV/AIDS	26 (7.4)	10 (14.5)	0.092
	HTN	140 (39.9)	20 (29.0)	0.117
LOS, days, median (IQR)	–	10 (6–17)	11 (4–27)	0.001

AIDS, acquired immunodeficiency syndrome; CKD, chronic kidney disease; DM, diabetes mellitus; HIV, human immunodeficiency virus; HTN, hypertension; IQR, interquartile range; LOS, length of stay.

The most common trigger for an RRS activation was altered mental status, followed by hypoxia and hypotension (Figure 2C). The 95 RRS activations involved 55 unique patients. Six patients had two RRS activations and 17 had three RRS activations during their hospital course. Figure 2D shows the percent distribution of the 95 RRS activations stratified by RRS event number for each patient and outcome (dead or alive) of the rapid response intervention.

Of the 55 first-time RRS activations, 42 (76%) patients survived the event. However, 35 (83%) patients who survived ultimately died during their hospital course. Interventions commonly included the addition or optimization of vasopressors (42%), intravenous fluid administration (42%) and oxygen supplementation (21%), (Table 2). None of the RRS activations resulted in a transfer to the designated ICU because no beds were available. Six patients were managed in the temporary ICU beds set up on the intervention units, as described in the Methods. This decision was made at the discretion of the rapid response team and based on availability of the continuous monitor. The family refused advanced ICU care on two occasions owing to financial constraints.

**TABLE 2:** Frequency of interventions during rapid response team activation.

Intervention	Frequency (% of rapid response events)
Oxygen support	20 (21%)
Intravenous fluid	40 (42%)
Vasopressor support	40 (42%)
Antibiotics	1 (< 1%)
Intubation	3 (< 1%)
ICU transfer.	0 (0%)
Blood transfusion	1 (< 1%)
Makeshift ICU with continuous monitoring	6 (< 1%)

ICU, intensive care unit.

Mortality rate was slightly lower in the control units during the investigation period, although our study was not adequately powered to determine a difference (Table 3).

**TABLE 3:** Admissions and mortality rates in control and intervention units from January 2019 Through July 2019.

Interventions units	Survival group	Mortality group (%)	*P*-value
**Interventions units**			
E5	228	56	0.729
A3	246	47	
Total	474	103 (21.7)	–
**Control units**			
A2	164	32	1.000
A4	187	37	
Total	351	69 (19.7)	–
Combined Total	997	172	–

## 4 Discussion

We successfully implemented an RRS at a resource-limited LMIC hospital (LUTH) through multidisciplinary engagement and staff organization, intense education, and initiation of improved communication structures, protocols, and critical care tools. To our knowledge, little has been published on such implementation in a resource-limited environment (20, 22). This program was overall well received and perceived to be of great benefit to the staff and patients at LUTH. Using a train-the-trainers model, this program is potentially scalable to units such as maternal medicine, the emergency department, and perhaps other teaching hospitals in the country (23, 24).

Many obstacles and limitations were encountered during this project that should be considered before expansion to other areas of the hospital or other institutions in resource- limited LMIC facilities. One of the primary and initial obstacles encountered was a delay in establishing a telephone system to standardize activation of the rapid response team members, which in turn delayed implementation of the rapid response program. Such a was a novel concept in Nigerian public hospitals, where everyone who has a personal mobile phone uses it in a decentralized manner for communication. However, that system was inadequate for implementing an RRS and required multiple consultations with different telecommunications companies to create a model that would work at the hospital. Centralized communication systems are ubiquitous in high-income countries, but establishing an effective, affordable, standardized method of activating the RRS team members can be quite difficult in LMICs.

A second obstacle was the perceived additional workload among both the nursing and house staff. Nurses are often overextended, especially during night shifts, because of the high number of high-acuity patients and high patient-to-nurse ratios, sometimes as high as one nurse to eight patients. During preparation for implementation, the nursing staff expressed concern over the perceived additional burden (especially documentation burden) on the already overtasked workforce. We addressed this concern by simplifying the documentation to one page. Although this change truncated the amount of data collected by event, it was a compromise that was necessary to promote documentation compliance.

A more challenging obstacle and limitation to address was the resistance from the medicine house staff to participate in what they perceived as a research project that was of minimal benefit to them. We addressed this concern in several meetings where the house staff were encouraged to view the quality improvement project as an opportunity for improving the care that they provided to patients who were already on their service, as well as the potential to improve outcomes and their hospital policy. Despite these efforts, internal medicine resident attendance at RRS activations remained substantially lower than that of the other team members. Improved strategies for motivation and engagement are necessary.

Other limitations and obstacles inherent to the healthcare systems in LMIC environments that we encountered included the common practice of requiring family members to purchase medications from the pharmacy before administration. Such systems do not exist in the hospitals and countries that have contributed nearly all of the data in support of RRS effectiveness. The delays from this type of pharmaceutical economic system severely impede the administration of potentially life-saving therapies during critical patient deteriorations and emergencies. Charging patients/families after the administration of indicated medications was a major culture change for LUTH and not without its associated financial risks should the patient and family be unable to pay. These financial hardships were also noted in the fact that in two of the RRS activations, the family refused escalation of care to the ICU because of costs. Cost considerations by the hospital and health system, as well as by the patients and their families, will remain a challenging barrier to success.

Notably, none of the patients who had an RRS activation were transferred to the ICU, though several met criteria. The literature reports different rates for ICU admission after an RRS activation (25), but most patients remain on the general unit after assessment and treatment by the rapid response team. We are unable to compare our findings with those of that body of literature, which is generated almost exclusively in countries and environments where ICU access is not limited or only minimally limited. LUTH, a 761-bed tertiary academic medical center, has only six ICU beds. In contrast, Johns Hopkins Hospital, a 960-bed institution, has 128 adult ICU beds, a ratio more typical of high-income countries. The severe limitation on ICU bed access in LMICs has implications for the implementation of RRS in those environments. There were 40 rapid response events that prompted the use of vasopressors, six of which utilized the temporary ICU-capable beds. This mobile ICU is a potential strategy for stabilizing patients and keeping them on the general unit.

This program helped highlight some of the deficiencies in the existing system, such as inadequate staffing and organization, inconsistent access to emergency supplies, and inadequacies of existing communication tools. Another important issue that was revealed was the weak infrastructure for identification and triage of terminally ill patients, resulting in minimal palliative care services and overuse of already scarce resources such as ventilators.

A final limitation is that this was a single-center study. However, we believe that LUTH is prototypical of most academic centers in Nigeria and perhaps in other LMICs and therefore a good model for what can be replicated at many institutions throughout Nigeria and sub-Saharan Africa.

## 5 Conclusion

Our study supports the hypothesis that the development and implementation of an RRS in an LMIC hospital is feasible. However, there is a need for more trained personnel and better infrastructure and standardized protocols for larger scale dissemination. Barriers such as physician engagement remain an impediment to full utilization. The documentation of RRS events and focused multidisciplinary review of each event can improve the quality of this intervention. Hospital buy-in and investment in staff and infrastructure can expand the impact of such programs throughout regional and national hospitals.

## Data Availability

The original contributions presented in this study are included in this article/supplementary material, further inquiries can be directed to the corresponding author.

## Footnotes

1
https://www.sccm.org/Education-Center/Educational-Programming/Fundamentals/Fundamental-Critical-Care-Support
